# The significance of the C-reactive protein to albumin ratio as a marker for predicting survival and monitoring chemotherapeutic effectiveness in patients with unresectable metastatic colorectal cancer

**DOI:** 10.1186/s40064-016-3529-y

**Published:** 2016-10-18

**Authors:** Masatsune Shibutani, Kiyoshi Maeda, Hisashi Nagahara, Yasuhito Iseki, Kosei Hirakawa, Masaichi Ohira

**Affiliations:** Department of Surgical Oncology, Osaka City University Graduate School of Medicine, 1-4-3 Asahi-machi Abeno–Ku, Osaka City, Osaka Prefecture 545-8585 Japan

**Keywords:** Colorectal cancer, Prognosis, Unresectable, C-reactive protein to albumin ratio, Chemotherapeutic effectiveness

## Abstract

Inflammation has been reported to play an important role in cancer progression and various inflammatory markers have been reported to be useful prognostic markers. The aim of this retrospective study was to evaluate the significance of the C-reactive protein to albumin (CRP/ALB) ratio in colorectal cancer patients who received palliative chemotherapy. We performed a retrospective review of 99 patients who underwent palliative chemotherapy for unresectable colorectal cancer between 2005 and 2010. The cutoff value of the CRP/ALB ratio was determined based on a receiver operating characteristics curve analysis. The relationship between the CRP/ALB ratio and survival was assessed. The cutoff value for the CRP/ALB ratio was 0.183. The high pretreatment CRP/ALB ratio group showed significantly worse overall survival. Patients with a high pretreatment CRP/ALB ratio and in whom the CRP/ALB ratio normalized after chemotherapy tended to have better overall survival than those in whom both the pretreatment and posttreatment CRP/ALB ratios were high. The CRP/ALB ratio is a useful marker for predicting survival and monitoring chemotherapeutic effectiveness in patients with unresectable metastatic colorectal cancer.

## Background

Colorectal cancer (CRC) is one of the most common causes of cancer-related death worldwide (Edwards et al. 2014). Unresectable metastasis is one of the most important prognostic factors for patients with CRC (Shibutani et al. 2015b). In spite of advances in chemotherapy, which include new cytotoxic and molecular targeted therapies, the prognosis of patients with unresectable metastatic CRC remains poor, with a median survival time of approximately 30 months (Heinemann et al. 2014; Grothey et al. 2008). The assessment of prognostic factors is therefore important for the management of unresectable metastatic CRC patients.

It is well known that there is a close relationship between inflammation and cancer progression (Mantovani et al. 2008; Balkwill and Mantovani 2001) and some inflammatory markers have been investigated as prognostic factors in CRC (Shibutani et al. 2013, 2014; McMillan et al. 2007; Maeda et al. 2015). The C-reactive protein-to-albumin (CRP/ALB) ratio has been reported to be a more accurate prognostic value in patients with various malignancies than the modified Glasgow prognostic score (mGPS) (Kinoshita et al. 2015; Xu et al. 2015), which is also calculated from the serum CRP and ALB concentration, and we previously reported on the prognostic significance of the CRP/ALB ratio in patients with CRC who underwent curative surgery (Shibutani et al. 2016). However, aside from our report, there have only been a few reports regarding the prognostic significance of the CRP/ALB ratio in patients with CRC (Ishizuka et al. 2016). Moreover, to the best of our knowledge, there are no published studies regarding the prognostic significance of the CRP/ALB ratio in patients with unresectable metastatic CRC. Therefore, we applied this marker to patients with unresectable metastatic CRC and compared the usefulness of the CRP/ALB ratio with that of other inflammatory markers for predicting and monitoring the therapeutic outcome.

The aim of the present study is to evaluate the significance of the CRP/ALB ratio as a marker for predicting survival and monitoring chemotherapeutic effectiveness in patients with unresectable metastatic CRC.

## Methods

### Patients

We retrospectively reviewed a database of 99 patients who underwent palliative combination chemotherapy for unresectable metastatic colorectal cancer at the Department of Surgical Oncology of Osaka City University between 2005 and 2010.

The patient characteristics are listed in Table 1. The patient population consisted of 57 males and 42 females, with a median age of 63 years (range 27–86). Forty of the patients had metachronous unresectable cancer; 59 had synchronous unresectable cancer. Fifty-four patients had single organ metastasis and 45 patients multiple organs affected by metastases. All of the patients underwent combination chemotherapy with oxaliplatin, or irinotecan plus 5-fluorouracil/leucovorin, or a prodrug of 5-fluorouracil as a first-line chemotherapy. Regimens considered to have the same efficacy were used for all of the patients in this study (Cassidy et al. 2008; Tournigand et al. 2004; Yamada et al. 2013). Sixty-five patients received 5-fluorouracil + leucovorin + oxaliplatin (FOLFOX), 21 patients received capecitabine + oxaliplatin (CapeOX), nine patients received 5-fluorouracil + leucovorin + irinotecan (FOLFIRI) and four patients received S-1 + oxaliplatin (SOX). Sixty-nine patients underwent chemotherapy combined with molecular targeted therapy. The median follow-up period for the surviving patients was 20.8 months (range 2.6–73.2 months). Sixty-three patients died during the follow-up period.

**Table 1:** The patients’ characteristics

Age (years)	
Median (range)	63 (27–86)
Gender	
Male	57
Female	42
Location of primary tumor	
Colon	57
Rectum	42
Histological type	
Well, moderately	78
Poorly, mucinous	12
Unknown	9
Detection of unresectable tumor	
Synchronous	59
Metachronous	40
The number of organs affected by metastasis	
One organ	54
Multiple organs	45
First-line chemotherapy regimen	
FOLFOX	65
CapeOX	21
FOLFIRI	9
SOX	4
Molecular targeted therapy	
Bevacizumab	50
Cetuximab	15
Panitumumab	4
None	30
The pretreatment C-reactive protein level	
Median (range)	0.32 (0.02–13.46)
The pretreatment albumin level	
Median (range)	3.9 (2.4–4.7)
The pretreatment CRP/ALB ratio	
Median (range)	0.084 (0.004–5.608)
The pretreatment NLR	
Median (range)	2.788 (0.580–16.306)
mGPS	
0	69
1	21
2	9

*FOLFOX* 5-fluorouracil + leucovorin + oxaliplatin, *CapeOX* capecitabine + oxaliplatin, *FOLFIRI* 5-fluorouracil + leucovorin + irinotecan, *SOX* S-1 + oxaliplatin, *CRP/ALB ratio* C-reactive protein to albumin ratio, *NLR* neutriphil to lymphocyte ratio, *mGPS* modified Glasgow prognostic score

### Evaluation

Response evaluations were performed every eight weeks. A variation of approximately one week was regarded as an allowable error. All of the patients were followed up with a physical examination, blood tests (these included measurements of tumor marker levels such as carcinoembryonic antigen [CEA]), computed tomography and ultrasonography.

The pretreatment blood samples were obtained within one week before the initiation of chemotherapy; the posttreatment blood samples were obtained eight weeks after the initiation of chemotherapy. The serum CRP and ALB concentrations were measured using a chemiluminescent immunoassay (Wako, Osaka, Japan) according to the manufacturer’s protocol. The differential white blood cell count was analyzed using an XE-5000 hematology analyzer (Sysmex, Kobe, Japan) based on the manufacturer’s protocol. The CRP/ALB ratio was calculated from the preoperative blood samples by dividing the serum CRP level by the serum ALB level. The mGPS was defined according to the methods of a previous report (Petrelli et al. 2015), using the combination of the serum CRP and ALB levels: patients with a CRP level of <1.0 mg/dl were allocated a score of 0; those in whom the CRP and ALB levels were ≥1.0 mg/dl and ≥3.5 g/dl, respectively, were allocated a score of 1; and those in whom the CRP and ALB levels were ≥1.0 mg/dl and ALB < 3.5 g/dl, respectively, were allocated a score of 2. The neutrophil to lymphocyte ratio (NLR) was calculated from a blood sample by dividing the absolute neutrophil count by the absolute lymphocyte count.

### Statistical analysis

The significance of the correlations between the pretreatment CRP/ALB ratio and the clinicopathological characteristics were analyzed using the *χ* ^2^ test. The duration of survival was calculated according to the Kaplan–Meier method. Differences in the survival curves were assessed using the log-rank test. A multivariate analysis was performed according to the Cox proportional hazard model. All of the statistical analyses were conducted using the SPSS software package for Windows (SPSS Japan, Tokyo, Japan). p values of <0.05 were considered to indicate statistical significance.

### Ethical consideration

This research was conformed to the provisions of the Declaration of Helsinki in 1975. All patients were informed of the investigational nature of this study and provided written informed consent. This retrospective study was approved by the ethics committee of Osaka City University.

## Results

### Classifications according to the pretreatment inflammatory markers

**Fig. 1:**
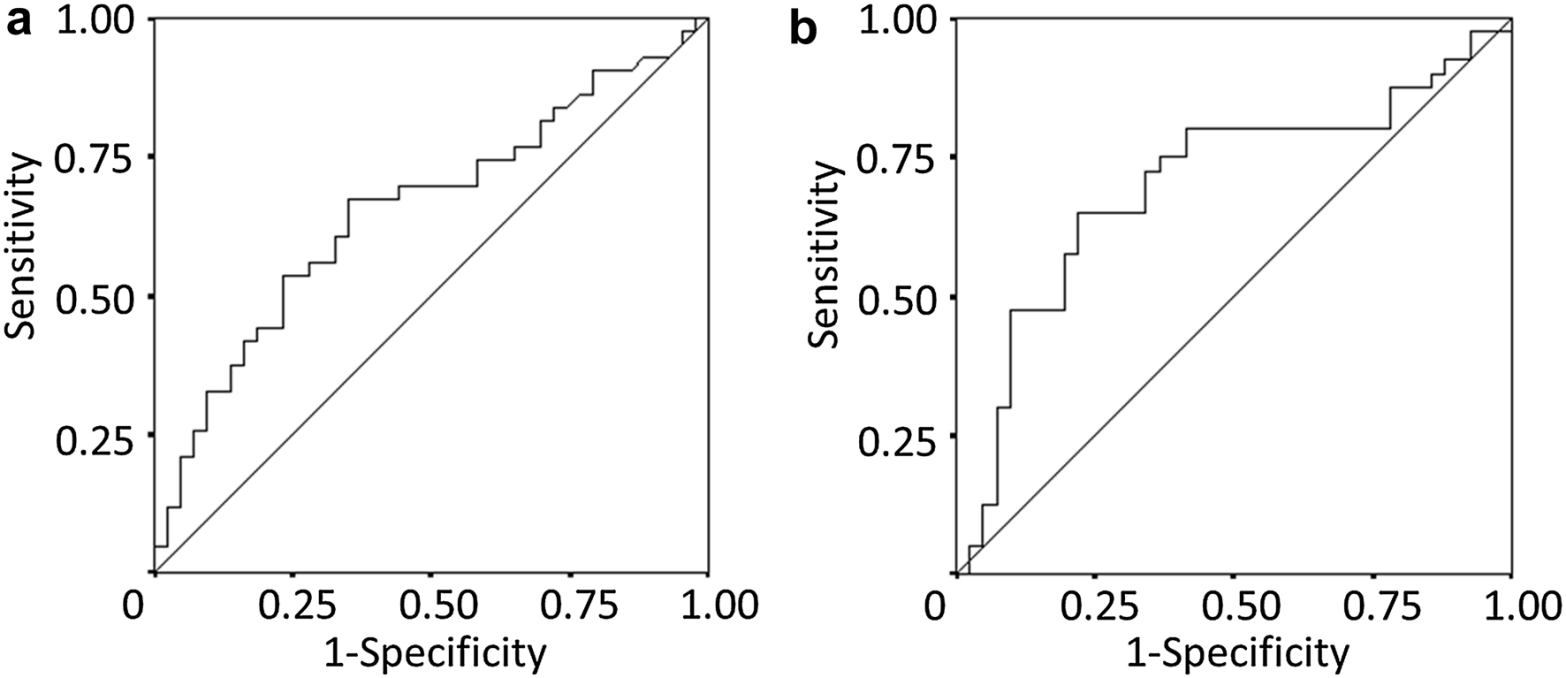
**a** A receiver operating characteristic curve analysis of the C-reactive protein to albumin (CRP/ALB) ratio in patients with unresectable metastatic colorectal cancer. Area under the curve = 0.655; 95 % confidence interval = 0.539–0.772; p = 0.013. **b** A receiver operating characteristic curve analysis of the neutrophil to lymphocyte ratio (NLR) in patients with unresectable metastatic colorectal cancer. Area under the curve = 0.701; 95 % confidence interval = 0.582–0.820; p = 0.002

We used the CRP/ALB ratio, which was a continuous variable, as the test variable and the 24-month survival (median survival time: 24 months) as the state variable. When we investigated the cut-off value for the CRP/ALB ratio using the receiver operating characteristic (ROC) curve, we found that the appropriate cut-off value for the CRP/ALB ratio was 0.183 (sensitivity: 53.5 %; specificity: 72.1 %) (Fig. 1a). We therefore set 0.183 as the cut-off value and the patients were classified into the high-CRP/ALB ratio (n = 36) and low-CRP/ALB ratio (n = 63) groups. Using the ROC curve in the same manner, we set the cut-off value for the NLR at 3.0 (sensitivity: 65.0 %, specificity: 78.0 %) (Fig. 1b). In accordance with the findings of previous reports, the study population was classified into the patients with an mGPS of 0 or 1 and the patients with an mGPS of 2 (Sugimoto et al. 2012; Furukawa et al. 2012).

### The correlations between the pretreatment CRP/ALB ratio and the clinicopathological factors

The correlations between the pretreatment CRP/ALB ratio and the clinicopathological factors are shown in Table 2. The pretreatment CRP/ALB ratio had no significant relationships with any of the clinicopathological factors except for the pretreatment mGPS and NLR.

**Table 2:** The correlations between the pretreatment CRP/ALB ratio and the clinicopathological factors

	Pretreatment CRP/ALB ratio		
	Low	High	*p* value
Gender			
Male	36	21	
Female	27	15	1.000
Age			
<65	34	19	
≥65	29	17	1.000
Location of primary tumor			
Colon	36	21	
Rectum	27	15	1.000
Detection of unresectable tumor			
Synchronous	34	25	
Metachronous	29	11	0.143
Histological type			
Well, moderately	51	27	
Poorly, mucinous	9	3	0.744
Peritoneal dissemination			
Negative	47	33	
Positive	16	3	0.061
The number of organs affected by metastasis			
One organ	36	18	
Multiple organs	27	18	0.534
Pretreatment CEA (ng/ml)			
≤5	9	4	
>5	53	31	0.765
Molecular targeted therapy			
No	17	12	
Yes	46	24	0.503
Pretreatment mGPS			
0	63	6	
1	0	21	
2	0	9	<0.001
Pretreatment NLR			
<3	41	13	
≥3	18	22	0.003

*CRP/ALB ratio* C-reactive protein to albumin ratio, *CEA* carcinoembryonic antigen, *mGPS* modified Glasgow prognostic score, *NLR* neutrophil to lymphocyte ratio

### The survival analysis based on the pretreatment inflammatory markers

**Fig. 2:**
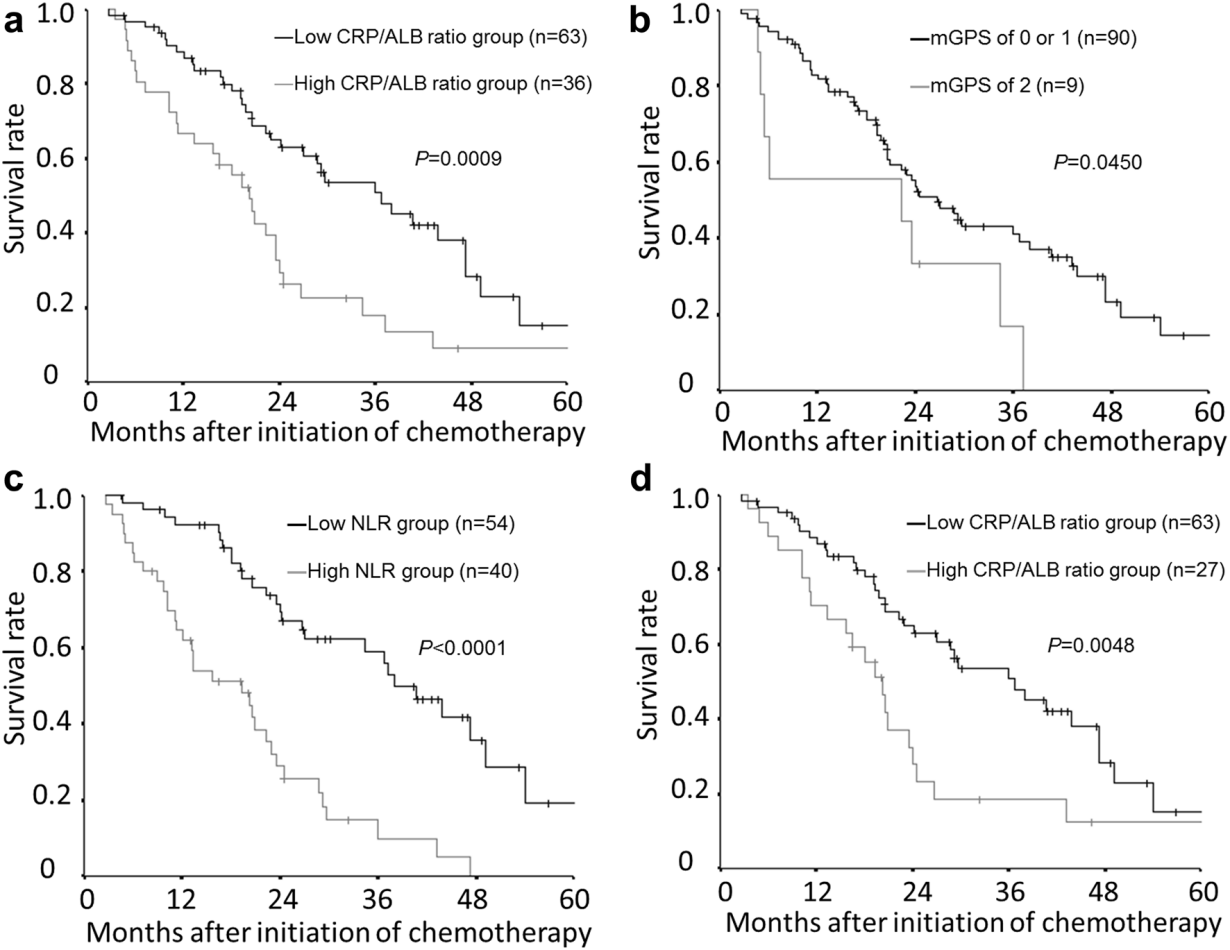
The Kaplan–Meier survival curves for overall survival. **a** The overall survival rate was significantly worse in the high-C-reactive protein to albumin (CRP/ALB) ratio group than in the low-CRP/ALB ratio group (p = 0.0009). **b** The overall survival rate was significantly worse in patients with a modified Glagow prognostic score (mGPS) of 2 than in those with an mGPS of 0 or 1 (p = 0.0450). **c** The overall survival rate was significantly worse in the high neutrophil to lymphocyte ratio (NLR) group than in the low NLR group (p < 0.0001). **d** Kaplan–Meier survival curves for overall survival in an analysis limited to the patients with a modified Glasgow prognostic score (mGPS) of 0 or 1. The overall survival rate was significantly worse in the high-C-reactive protein to albumin (CRP/ALB) ratio group than in the low-CRP/ALB ratio group (p = 0.0048)

The overall survival rate was significantly worse in the high pretreatment CRP/ALB ratio group than in the low pretreatment CRP/ALB ratio group (p = 0.0009) (Fig. 2a). The overall survival rate was significantly worse in patients with an mGPS of 2 than in those with an mGPS of 0 or 1 (p = 0.0450) (Fig. 2b). The overall survival rate was significantly worse in the high neutrophil to lymphocyte ratio group than in the low neutrophil to lymphocyte group (p < 0.0001) (Fig. 2c).

### The prognostic factors influencing long-term survival

The correlations between overall survival and the various clinicopathological factors are shown in Table 3. According to the results of a univariate analysis, overall survival showed significant relationships with the number of organs affected by metastasis (p = 0.024), the use of a molecular targeted therapy (p = 0.001), the pretreatment CRP/ALB ratio (p = 0.001) and the pretreatment NLR (p < 0.001). A multivariate analysis indicated that the use of a molecular targeted therapy (hazard ratio 0.341; 95 % confidence interval 0.186–0.626; p = 0.001), the pretreatment CRP/ALB ratio (hazard ratio 1.866; 95 % confidence interval 1.057–3.295; p = 0.031) and the pretreatment NLR (hazard ratio 2.706; 95 % confidence interval 1.483–4.939; p = 0.001) were independent prognostic factors for overall survival.

**Table 3:** The correlations between overall survival and various clinicopathological factors

	Univariate analysis			Multivariate analysis		
	Hazard ratio	95 % CI	p value	Hazard ratio	95 % CI	p value
Gender (female vs. male)	1.473	0.895–2.423	0.128			
Age (≥65 vs. <65)	1.492	0.907–2.454	0.115			
Location of primary tumor (colon vs. rectum)	1.273	0.769–2.108	0.348			
Detection of unresectable tumor (synchronous vs. metachronous)	1.595	0.946–2.688	0.080	1.099	0.595–2.030	0.763
Histological type (poorly, mucinous vs. well, moderately)	1.417	0.688–2.916	0.344			
Peritoneal dissemination (yes vs. no)	1.124	0.609–2.075	0.708			
The number of organs affected by metastasis (≥2 vs. <2)	1.775	1.078–2.923	0.024	1.115	0.622–1.997	0.715
Pretreatment CEA (>5 ng/ml vs. ≤5 ng/ml)	2.193	0.940–5.113	0.069	1.370	0.526–3.571	0.520
Molecular targeted therapy (yes vs. no)	0.391	0.227–0.676	0.001	0.341	0.186–0.626	0.001
Pretreatment CRP/ALB ratio (>0.183 vs. ≤0.183)	2.301	1.390–3.807	0.001	1.866	1.057–3.295	0.031
Pretreatment NLR (>3 vs. ≤3)	3.777	2.191–6.511	<0.001	2.706	1.483–4.939	0.001

*CEA* carcinoembryonic antigen, *CRP/ALB ratio* C-reactive protein to albumin ratio, *NLR* neutrophil to lymphocyte ratio

### The survival analysis based on the pretreatment CRP/ALB ratio, limited to the patients with an mGPS of 0 or 1

We then performed a sub-analysis limited to patients with an mGPS of 0 or 1. Among these patients, the overall survival rate was significantly worse in the high-CRP/ALB ratio group than in the low-CRP/ALB ratio group (p = 0.0048) (Fig. 2d).

### The correlation between the normalization of the CRP/ALB ratio at eight weeks after chemotherapy and survival

**Fig. 3:**
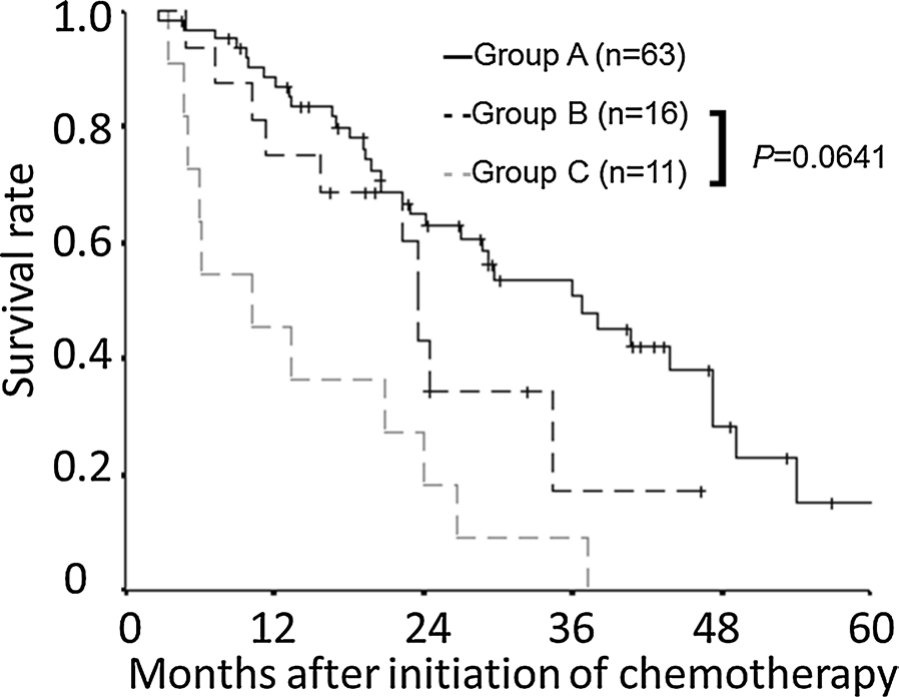
Overall survival according to the combination of the pretreatment and posttreatment C-reactive protein to albumin (CRP/ALB) ratio values. The prognosis of the patients in group B tended to be better than that in the patients of group C (p = 0.0641)

We evaluated the prognostic significance of the normalization of the CRP/ALB ratio at eight weeks after the initiation of chemotherapy. We categorized the patients into three groups according to their pretreatment and posttreatment CRP/ALB ratio values. Patients with a low pretreatment CRP/ALB ratio were categorized into group A. Patients with a high pretreatment CRP/ALB ratio and a normalized CRP/ALB ratio at eight weeks after the initiation of chemotherapy were categorized into group B. Patients with high pretreatment and posttreatment CRP/ALB ratio values were categorized into group C. The patients in group B tended to exhibit a better prognosis than those in group C (p = 0.0641) (Fig. 3).

## Discussion

There have been various studies on the close relationship between inflammation and cancer since the relationship was first reported by Virchow in 1863 (Mantovani et al. 2008; Balkwill and Mantovani 2001). Recently, inflammation has been widely recognized to contribute to cancer progression and various inflammatory markers, such as the NLR, CRP, GPS and mGPS have been reported to be correlated with the survival of patients with CRC (Shibutani et al. 2013, 2014; McMillan et al. 2007; Sugimoto et al. 2012; Furukawa et al. 2012).

Inflammation induces an increase in the levels of cytokines, which play an important role in tumor proliferation, progression, invasion and metastasis, as well as in resistance to chemotherapy (Colotta et al. 2009; Coussens and Werb 2002; Heikkilä et al. 2007). Therefore, inflammatory markers are considered to reflect the momentum of cancer growth. Furthermore, the levels of systemic inflammatory markers have been reported to correlate with tumor volume (Shibutani et al. 2015b). In this way, inflammatory markers are considered to be useful for predicting and monitoring the therapeutic outcome in patients with malignancies.

The CRP/ALB ratio was primarily investigated for the purpose of predicting mortality in patients with sepsis. The CRP/ALB ratio was also applied to predicting the prognosis of patients with cancer and its usefulness has been reported in patients with various cancers, such as hepatocellular carcinoma, esophageal cancer and lung cancer (Kinoshita et al. 2015; Xu et al. 2015; Zhou et al. 2015). In previous reports, the CRP/ALB ratio was shown to have an outstanding prognostic value in comparison to other established inflammation-based prognostic markers (Kinoshita et al. 2015; Xu et al. 2015). Although the area under curve (AUC) value of the CRP/ALB ratio was lower than that of the NLR, the CRP/ALB ratio was shown to be a valuable marker and was thus found to be sufficient for predicting survival in the present study.

As Casadei Gardini reported, the serum CRP concentration alone is also significantly correlated with the survival (Casadei Gardini et al. 2016). We came to the same conclusion based on the findings in our previous study (Shibutani et al. 2015b). However, Ranzani reported that the CRP/ALB ratio was more closely correlated with survival than the CRP values alone (Ranzani et al. 2013). By combining the CRP and albumin values, which have both been reported to be independent prognostic factors for various types of cancer, the CRP/ALB ratio is believed to be a more useful marker.

The mGPS, which has been reported to be a useful prognostic marker in patients with CRC, is similar to the CRP/ALB ratio, as both results can be calculated from the serum CRP and albumin concentration. However, the majority of the patients are classified with an mGPS of 0 or 1, which is associated with a better prognosis (Sugimoto et al. 2012; Furukawa et al. 2012). In the present study, approximately 90 % of the patients were found to have an mGPS of 0 or 1. For this reason, most patients could not be classified by the mGPS. On the other hand, the patients who had an mGPS of 0 or 1 could be classified into two groups according to their CRP/ALB ratios and significant differences were observed between the two groups. It can therefore be said that the CRP/ALB ratio is a more accurate prognostic marker than the mGPS.

Moreover, the normalization of the CRP/ALB ratio at eight weeks after the initiation of chemotherapy tended to be correlated with an improvement in overall survival. Based on this result, the CRP/ALB ratio is considered to be a useful marker for monitoring the effectiveness of chemotherapy as well as for predicting survival. On the other hand, it has been reported that there is no relationship between the normalization of the NLR and survival, because the value is easily affected by chemotherapy-induced myelosuppression (Shibutani et al. 2015a). Therefore, the CRP/ALB ratio was believed to be superior to the NLR for evaluating the efficacy of chemotherapy. Although previous studies have primarily focused on the prognostic significance of the pretreatment CRP/ALB ratio, in the present study, the CRP/ALB ratio was revealed to be a useful marker for monitoring the effectiveness of chemotherapy as well as for predicting survival.

Although we set 0.183 as the CRP/ALB ratio cut-off value according to the results of the ROC analysis, other studies have used various cut-off values. Because the inflammatory markers were reported to be associated with the degree of tumor progression, such as the TNM stage (Shibutani et al. 2013; Xu et al. 2015; Ishizuka et al. 2016), the appropriate cut-off value may change according to the background characteristics of the patients.

The present study is associated with some possible limitations. First, we evaluated a relatively small number of patients and the study design was retrospective in nature. Second, comorbidities that may have affected systemic inflammation and the serum albumin concentration (such as infection, ischemia, acute coronary disease, liver cirrhosis, and nephrotic syndrome) were not taken into consideration. A large prospective study should therefore be performed to confirm our findings.

## Conclusions

The CRP/ALB ratio is a useful marker not only for predicting survival, but also for monitoring chemotherapeutic effectiveness in patients with unresectable metastatic colorectal cancer who receive palliative chemotherapy.

## Abbreviations

CRP: C-reactive protein
ALB: albumin
mGPS: modified Glasgow prognostic score
FOLFOX: 5-fluorouracil + leucovorin + oxaliplatin
CapeOX: capecitabine + oxaliplatin
FOLFIRI: 5-fluorouracil + leucovorin + irinotecan
SOX: S-1 + oxaliplatin
CEA: carcinoembryonic antigen
NLR: neutrophil to lymphocyte ratio
ROC: receiver operating characteristic
